# Characterization and manipulation of the bacterial community in the midgut of *Ixodes ricinus*

**DOI:** 10.1186/s13071-022-05362-z

**Published:** 2022-07-09

**Authors:** Melina Garcia Guizzo, Kristyna Dolezelikova, Saraswoti Neupane, Helena Frantova, Alena Hrbatova, Barbora Pafco, Jessica Fiorotti, Petr Kopacek, Ludek Zurek

**Affiliations:** 1 Central European Institute of Technology (CEITEC), Center for Infectious Diseases and Microbiology, University of Veterinary Sciences, Brno, Czech Republic; 2 Biology Centre, Institute of Parasitology, Czech Academy of Sciences, Ceske Budejovice, Czech Republic; 3 Department of Entomology, Kansas State University, Manhattan, KS USA; 4 Institute of Vertebrate Biology, Czech Academy of Sciences, Brno, Czech Republic; 5 Department of Chemistry and Biochemistry, Mendel University, Brno, Czech Republic; 6 Department of Microbiology, Nutrition and Dietetics, Czech University of Life Sciences, Prague, Czech Republic

**Keywords:** *Ixodes ricinus*, Midgut, Microbiome, Culturing, High-throughput sequencing, Capillary feeding, Microbiome manipulation

## Abstract

**Background:**

Ticks are obligate hematophagous arthropods transmitting a wide range of pathogens to humans and animals. They also harbor a non-pathogenic microbiota, primarily in the ovaries and the midgut. In the previous study on *Ixodes ricinus*, we used a culture-independent approach and showed a diverse but quantitatively poor midgut bacterial microbiome. Our analysis also revealed the absence of a core microbiome, suggesting an environmental origin of the tick midgut microbiota.

**Methods:**

A bacterial analysis of the midgut of adult females collected by flagging from two localities in the Czech Republic was performed. Using the culture-independent approach, we tested the hypothesis that the midgut microbiome is of the environmental origin. We also cultured indigenous bacteria from the tick midgut and used these to feed ticks artificially in an attempt to manipulate the midgut microbiome.

**Results:**

The midgut showed a very low prevalence and abundance of culturable bacteria, with only 37% of ticks positive for bacteria. The culture-independent approach revealed the presence of *Borrelia* sp., *Spiroplasma* sp., *Rickettsia* sp., *Midichloria* sp*.* and various mainly environmental Gram-positive bacterial taxa. The comparison of ticks from two regions revealed that the habitat influenced the midgut bacterial diversity. In addition, the midgut of ticks capillary fed with the indigenous *Micrococcus luteus* (Gram-positive) and *Pantoea* sp. (Gram-negative) could not be colonized due to rapid and effective clearance of both bacterial taxa.

**Conclusions:**

The midgut microbiome of *I. ricinus* is diverse but low in abundance, with the exception of tick-borne pathogens and symbionts. The environment impacts the diversity of the tick midgut microbiome. Ingested extracellular environmental bacteria are rapidly eliminated and are not able to colonize the gut. We hypothesize that bacterial elimination triggered in the midgut of unfed adult females is critical to maintain low microbial levels during blood-feeding.

**Graphical Abstract:**

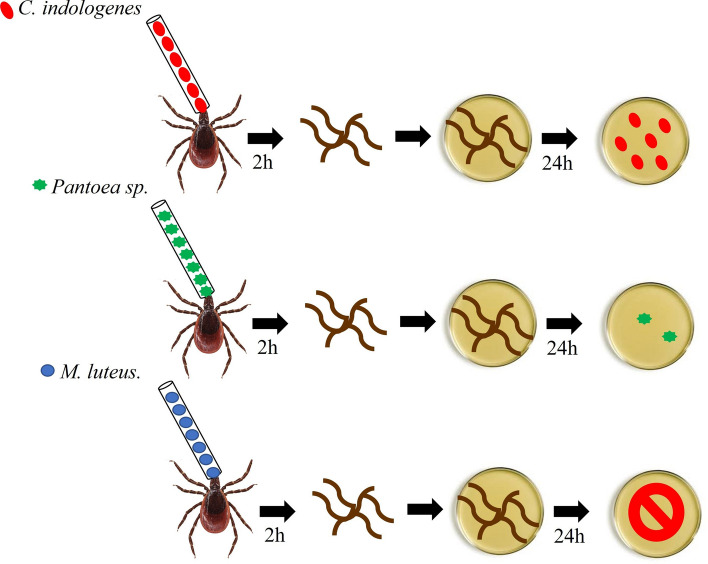

**Supplementary Information:**

The online version contains supplementary material available at 10.1186/s13071-022-05362-z.

## Background

It is well established that hematophagous arthropods harbor an abundant microbiome that is primarily located in the gut. Blood is a rich source of nutrients, and its injestion can lead to the expansion of the gut microbial community by several orders of magnitude [1–4]. An abundant microbial community may play a role in the host metabolism, development, nutrition and reproduction [5]. Diversity of the gut microbiome can be influenced by the habitat, diet, temperature, sex and several other factors shaping complex host-microbiome interactions [6, 7]. The native gut microbiota may also interact with pathogens that arthropods vector in neutral, detrimental or beneficial relationships. Natural or artificially induced detrimental interactions have been suggested as a promising strategy to reduce the vector competence of blood-feeding arthropods for the pathogens they carry [2, 8, 9].

Ticks are obligate hematophagous arthropods that feed on a wide range of hosts. They harbor mutualist symbionts, commensal microorganisms and human and animal pathogens [10–13]. Tick-borne pathogens, such as *Borrelia* sp., typically persist and/or multiply in the tick midgut before they are transmitted further [14]. While evading host immune responses, pathogens interact with non-pathogenic microbes in the midgut, and this interaction may impact their colonization and transmission [15–17]. Metagenomic studies on the tick midgut showed that this organ carries a diverse bacterial community represented by numerous operational taxonomic units (OTUs) [18–24]. However, in general, the bacterial abundance in the tick midgut is low, especially when assessed by culturing methods [25–31], confirming the lack of a core gut microbiome. In our previous studies, we showed that the large microbial diversity in the midgut of *Ixodes ricinus* contrasts with a low abundance, declining from 10^4^ bacteria/midgut in unfed females to 10^2^ bacteria/midgut in fully fed females [32]. Our studies also revealed the absence of a core microbiome, indicating an environmental origin and a transient nature of the midgut microbiota of *I. ricinus* [32] and *Amblyomma americanum* [33]. While pathogens of most obligate blood-feeders, such as mosquitoes and kissing bugs, interact with numerous indigenous microbes in the digestive tract [3, 34], the midgut of *I. ricinus* represents an environment with a very small microbial community, which reduces any potential natural detrimental interactions between pathogens and indigenous microbiota. However, the manipulation of *I. ricinus* midgut microbiome might be exploited as a promising strategy for reducing tick vector competence for pathogens [35–37].

In this study, we coupled the culture-independent method with the culturing approach and analyzed the midgut microbiome of questing females of *I. ricinus* collected from two regions in the Czech Republic. This allowed us to test the hypothesis of the environmental origin of the tick midgut microbiota. In addition, we artificially fed adult female ticks with *Pantoea* sp. and *Micrococcus luteus* isolated from the tick midgut in an attempt to manipulate the midgut microbiome.

## Methods

### Culture-dependent bacterial quantification and identification

Unfed *I. ricinus* females were collected by flagging on grass from an urban park in the city of Brno (*n* = 38) in Southern Moravia, and from a grass forest where there is relatively low human activity near the city of Ceske Budejovice (*n* = 43) in Southern Bohemia, both sites located in the Czech Republic. Captured ticks were surface sterilized with 0.05% sodium hypochlorite (commercial bleach; SAVO brand, Unilever Czech Republic, Prague, Czech Republic) for 3 min, followed by 70% ethanol for 1 min and then three washes in sterile potassium buffered saline (PBS) (Sigma-Aldrich, St. Louis, MO, USA) to prevent any body surface contamination [38, 39]. Individual whole midguts were dissected out and homogenized in 200 µl of PBS (Sigma-Aldrich). One half of each homogenate was spread-plated on 5.0% sheep blood-agar (Oxoid, Basingstoke, UK) and incubated aerobically at 26 °C for 72 h. Colony-forming colonies (CFUs) were counted and re-calculated into CFUs per tick. Morphologically distinct colonies were picked and streaked on TSA (Tryptic Soy Agar; Sigma-Aldrich) and incubated at 26 °C for identification. The other half of each homogenate was kept at−80 °C for further culture-independent analysis.

Bacterial identification was done using matrix-assisted laser desorption/ionisation–time-of-flight mass spectrometry (MALDI-TOF MS) on the Microflex LT bench-top MALDI-TOF mass spectrometer (Bruker Daltonik GmbH & Co. KG, Bremen, Germany), as described previously [40]. Briefly, the bacterial culture was placed on the MALDI plate, overlaid with 1.0 μl of the matrix solution containing 10 mg/ml HCCA (a-cyano-4-hydroxycinnamic acid; Sigma-Aldrich) dissolved in 50% acetonitrile (Sigma-Aldrich) and 2.5% trifluoroacetic acid and then air-dried. The mass spectra were processed using the MALDI Biotyper 3.0 software package (Bruker Optik GmbH, Leipzig, Germany) containing 6903 reference spectra. Identification was performed according to the criteria recommended by the manufacturer.

### Culture-independent bacterial identification

One half of each midgut homogenate was used for genomic DNA extraction and isolation using the PowerSoil DNA isolation kit (Qiagen, Hilden, Germany) according to the manufacturer’s instructions. To increase the specificity of DNA amplification, a pre-PCR was performed using the primer pairs 8F (5′-AGAGTTTGATCCTGGCTCAG-3′) and 907R (5′-CCGTCAATTCMTTTRAGTTT-3′) of the* 16S* ribosomal RNA (rRNA) gene. Illumina library preparation and subsequent sequencing were performed as previously described [41]. Briefly, the V3-V4 region of the* 16S* rRNA gene was amplified using the primer pairs 341F (5′-CCTACGGGAGGCAGCAG-3′) and 805R (5′-GACTACHVGGGTATCTAATCC-3′). The sequencing library was generated using a two-step-PCR approach following the Nextera primer design for Illumina. Analysis was carried out in two technical replicates. The library was sequenced using the MiSeq Reagent kit v2 (2X250 bp pair-end reads) for the Illumina MiSeq platform (Illumina, Inc., San Diego, CA, USA).

### Data analysis

All data were analyzed in the R statistical programming environment (version 3.6.2; R Foundation for Statistical Computing, Vienna, Austria) using packages stats [42], phyloseq [43], vegan [44], ape [45] and ggplot2 [46]. The erroneous OTUs with low abundance (< 0.005% of total abundance) [47] and contaminant OTUs present in the sterile water sample (negative control) were removed from the OTU data set. The abundance of OTUs was used to generate a rarefaction curve for estimating the species richness in individual tick midguts. Alpha diversity indices, including species richness, Shannon diversity index and Faith’s phylogenetic diversity (Faith’s PD), were estimated in the Vegan (version 2.5-6) and Ape (version 5.0) packages [44, 45]. To determine if there were significant differences between the group means of tick midgut bacterial alpha-diversity between localities (Brno and Ceske Budejovice), Wilcoxon rank sum test was performed. Bacterial community composition in each sample was compared using principal coordinate analysis (PCoA). Briefly, a Bray-Curtis dissimilarity index was used to calculate PCoAs, and the first two axes of PCoA were plotted to visualize the bacterial community composition in each sample using the ggplot2 package [46]. Permutational multivariate analysis of variance (Adonis) was used to examine if there was a statistical difference in bacterial community composition between two localities.

Bacterial OTUs that had same taxonomic lineages were grouped at the phylum and genus level. The distribution of bacterial phyla in each sample was visualized in a bar plot. A Wilcoxon rank sum test was used to determine a significant difference in the mean relative abundance of each phylum between localities. Difference in the mean relative abundance of each genus between two localities was also determined by the Wilcoxon rank sum test. Further, the prevalence of each of those 50 genera in the two localities was also visualized in the heatmap. The abundance of CFUs (log transformed) of cultured bacterial genera and the most abundant bacterial relative abundance genera in each sample were visualized in a bar plot. All statistical tests with *P*-value < 0.05 were considered to be statistically significant.

### Tick capillary feeding

Wild unfed *I. ricinus* females were glass capillary fed with either *Pantoea* sp. or *M. luteus* isolated from the tick midgut as described above. As a positive control for bacterial ingestion*,* we used *Chryseobacterium indologenes* [48]. Ten females were fed in each experiment for 2 h at 37 °C in a humid chamber with the bacterial suspension of OD600 = 0.1 for *M. luteus*, *Pantoea* sp. and *C. indologenes*. The number of CFUs offered to and ingested by the ticks were calculated based on CFU counts in 1.0 µl of the bacterial suspension and the conversion of the volume ingested into microliters of the bacterial suspension, respectively. After 2 h of feeding on the bacterial suspension, ticks were surfaced-sterilized as described above. The midgut of ticks fed with *M. luteus*, *Pantoea* sp. or *C. indologenes* was dissected out and homogenized individually in 120 µl of PBS using sterile pestles. A 100-µl aliquot of each homogenate was spread-plated on TSA and allowed to grow overnight at 30 °C. Total CFUs were counted and recalculated in CFUs per tick midgut. The colony morphology was used to distinguish *M. luteus*, *Pantoea* sp. and *C. indologenes* from the background of other culturable bacteria.

To visualize bacterial cells in the midgut by microscopy, ticks were fed with green fluorescent protein (GFP)-labeled *Escherichia coli* DH5α with the plasmid pGFPuv (Clontech, Mountain View, CA, USA) or GFP-labeled *Staphylococcus aureus* RN6390 strain ALC1743 with plasmid psk236, as described above, with an OD600 = 1.0. Ticks were then dissected in sterile PBS, the dorsal cuticle was carefully removed and the tick internal organs were fixed in situ in 4% formaldehyde for 2.5 h at room temperature. The midgut was transferred and mounted in DABCO and examined under the fluorescence microscope (Olympus model BX3 light microscope; Olympus Corp., Tokyo, Japan).

## Results

### Bacterial community assessed by the culturing approach

**Fig. 1:**
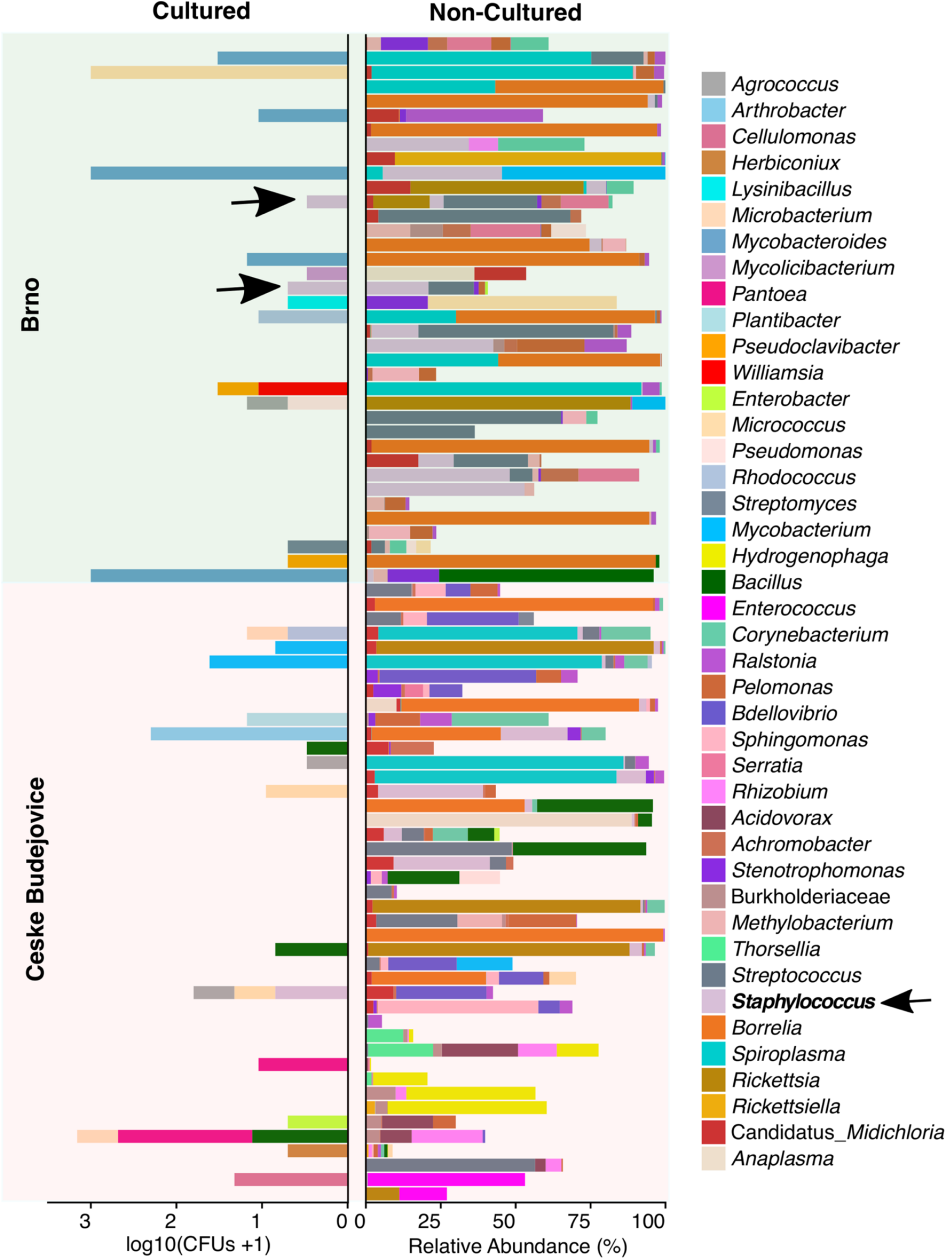
Bacteria identified by culturing and culture-independent methods in the midgut of individual *Ixodes ricinus* adult females collected from Brno and Ceske Budejovice. Bacterial CFUs were log transformed. Bacterial genera from the culture-independent method are represented by the relative abundance (%). Black arrows show an example of the bacterial taxon detected in individual ticks by both methods. Abbreviations: CFU, Colony-forming units

The culturing approach for detection of bacteria in the midgut of unfed *I. ricinus* females revealed very low bacterial abundance. Of 81 samples, bacterial isolates were cultured from 30 (37.0%) ticks, with variable abundance that ranged from 2 to 1000 bacteria/midgut (median: 9). Isolates were representative 20 genera and 32 species (Fig. 1). The most abundant (log_10_ CFU = 3) and prevalent was *Mycobacteroides salmoniphilum* isolated from five ticks from Brno only. The second most abundant species was *Micrococcus yunnanensis* (log_10_ CFU = 3) but isolated from only one tick. Detected taxa also included *Bacillus* sp., *Mycobacterium* sp., *Staphylococcus* sp., *Staphylococcus epidermidis*, *M. luteus*, *Rhodococcus* sp., *Pantoea* sp., *Pseudomonas* sp. and *Enterobacter* sp. (Fig. 1).

### Bacterial community assessed by culture-independent approach

**Fig. 2:**
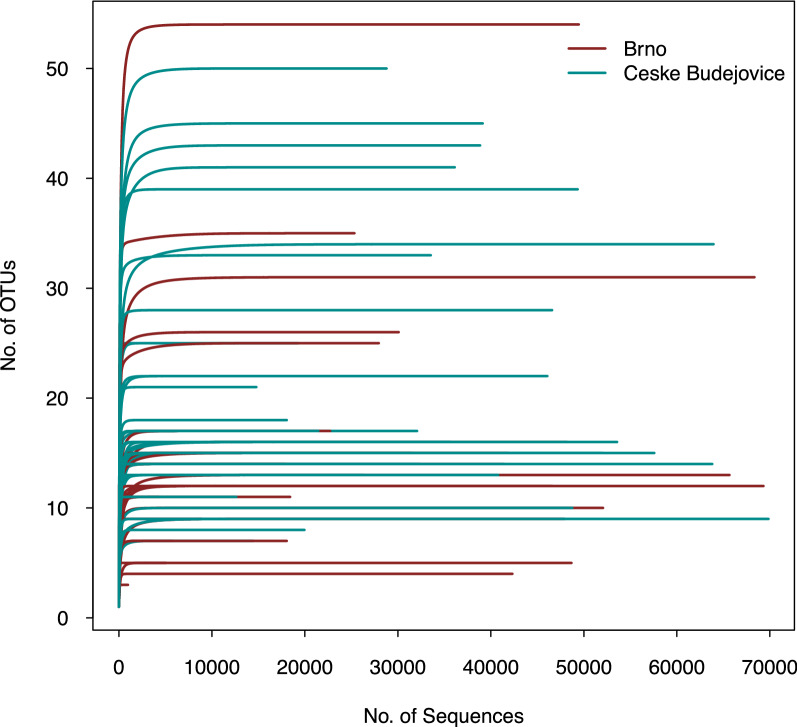
Rarefaction curves of* 16S* rDNA sequences in the midgut of *Ixodes ricinus*

**Fig. 3:**
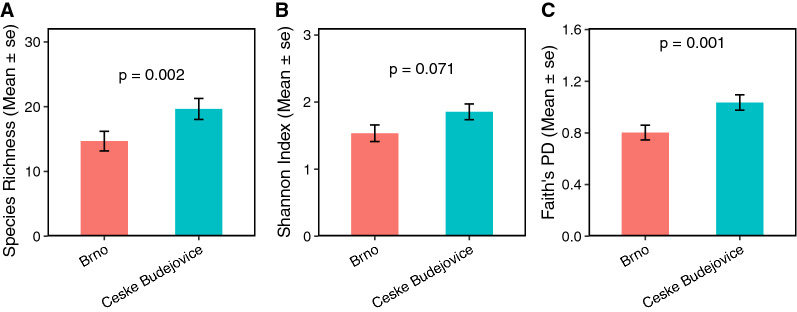
Effect of location on bacterial species richness (**a**), Shannon diversity index (**b**) and Faith’s PD (**c**) of the midgut bacterial community of *Ixodes ricinus*. Abbreviations: se, Standard error

**Fig. 4:**
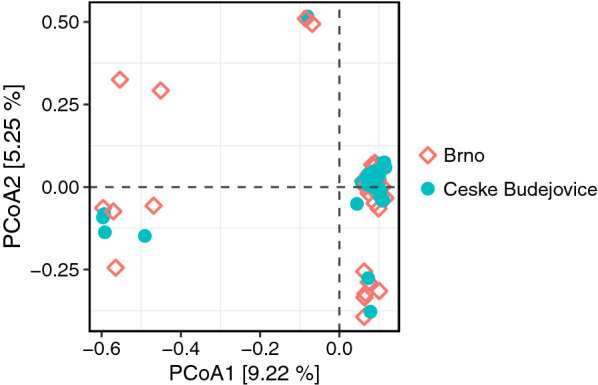
Bacterial community composition in individual tick midguts. PCoA plots generated using the Bray-Curtis index. Abbreviations: PCoA, Principal coordinate analysis

In total, 2,569,085 sequence reads were clustered into 799 OTUs. Of the total sequences, 43.7% (1,123,359) sequence reads were from Brno and 56.3% (1,445,726) sequence reads were from Ceske Budejovice. The plateau of the rarefaction curves indicated sufficient sequencing depth with an adequate representation of microbial communities (Fig. 2). Overall, bacterial species richness (observed OTUs) in the individual tick midgut ranged from 3 to 54. The Shannon diversity index ranged from 0.18 to 3.21, and Faith’s PD ranged from 0.28 to 2.16. The Shannon diversity index varied non-significantly between the two localities (*P* = 0.071; Fig. 3b). However, both species richness (*P* = 0.002) and Faith’s PD (*P* = 0.001) were significantly different between the two localities (Fig. 3a, c). Bacterial community composition in the individual tick midgut varied, but there was no significant difference between Brno and Ceske Budejovice (Fig. 4).

**Fig. 5:**
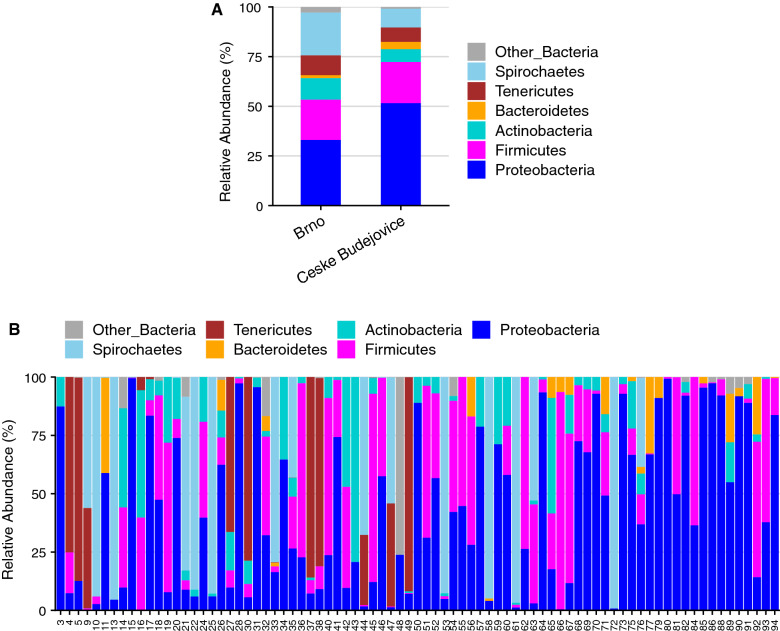
Mean bacterial relative abundance (%, at phylum level) associated with the geographic location (**a**) and in individual tick midgut samples (**b**). Sequences that were classified as “Bacteria_unclassified” and phyla with low abundance were grouped into Other_Bacteria

The most prevalent (98.77% of tick samples) and abundant phylum was Proteobacteria (Fig. 5a) followed by Firmicutes, Bacteroidetes, Actinobacteria, Spirochetes and Tenericutes (Fig. 5a). The relative abundance of those phyla varied across individual tick midguts (Fig. 5b). Interestingly, there was a significant difference between the mean relative abundance of Proteobacteria (*P* = 0.009), Bacteroidetes (*P* = 0.003) and Spirochaetes (*P* = 0.021) between two localities.

**Fig. 6:**
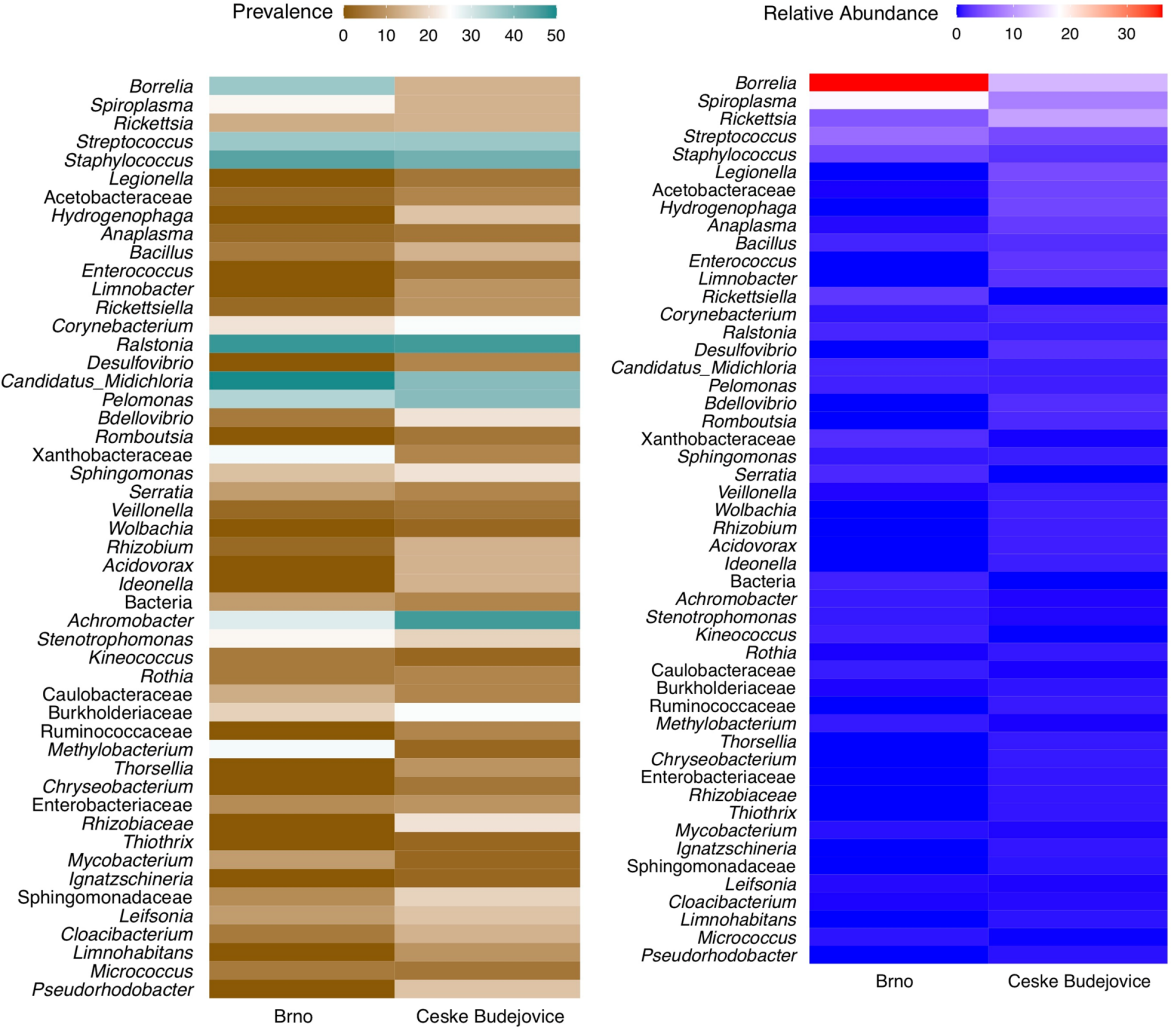
Mean relative abundance and prevalence of 50 most abundant taxa in the midgut of *Ixodes ricinus* adult females detected by the culture-independent method

Overall, using the culture-independent method, we detected a total of 205 genera. Among these, several genera had a relatively high abundance and prevalence, such as *Borrelia* (22.8% of total sequences, in 24.7% of ticks), *Spiroplasma* (12.2%, in 18.5% of ticks), *Rickettsia* (8.2%, in 13.6% of ticks), *Streptococcus* (5.1%, in 37.0% of ticks), *Staphylococcus* (2.8%, in 43.2% of ticks), *Midichloria* (1.2%, in 44.4% of ticks), *Ralstonia* (1.2%, in 46.9% of ticks), *Pelomonas* (1.2%, in 37.0% of ticks) and *Achromobacter* (0.6%, in 38.3% of ticks). The 50 most abundant genera represented 88.72% (2,279,269 sequence reads) of all sequences (Fig. 6; Additional file 1: Table S1**).** The abundance of genus *Borrelia* varied significantly (*P* = 0.02) between Brno (38.8% ticks) and Ceske Budejovice (13.9% ticks) (Fig. 6). Also, *Midichloria* sp. was highly prevalent **(**50% of ticks in Brno, and 40% of ticks in Ceske Budejovice, Fig. 6), but with low abundance in both localities.

### Common bacterial genera in both culture and non-cultured methods

We detected several genera using both the culture and culture-independent methods, including *Bacillus*, *Staphylococcus*,* Streptomyces*,* Micrococcus*, *Rhodococcus*,* Mycobacterium*,* Pseudomonas* and *Enterobacter* (Fig. 1). The relative abundance of the most common genera detected by the culture-independent method was very low (*Bacillus* [mean abundance {m.a.}]: 1.7%; range: 0–71.6% per tick), *Enterobacter* (m.a: 0.03%; range: 0–1.8% per tick), *Micrococcus* (m.a: 0.3%; range: 0–63.0% per tick), *Mycobacterium* (m.a: 0.4%; range: 0–64.6% per tick), *Pseudomonas* (m.a: 0.21%; range: 0–13.5% per tick), *Rhodococcus* (m.a: 0.02%; range: 0–1.4% per tick), *Staphylococcus* (m.a: 2.8%; range: 0–52.9% per tick) and *Streptomyces* (m.a: 0.04%; range: 0–5.1% per tick)].

Several bacterial taxa, including *Borrelia* (abundance range: 0–99.3% per tick), *Rickettsia* (abundance range: 0–92.6% per tick), *Spiroplasma* (abundance range: 0–91.8% per tick) and *Midichloria* (abundance range: 0–17.5% per tick) were detected, as expected, by the culture-independent method only, with a comparatively high prevalence in both localities (Fig. 1). On the other hand, using the culturing approach, we detected *Mycobacteroides* sp. in Brno only, where it was relatively highly prevalent and abundant. *Pantoea* sp. and *Bacillus* sp. were detected in Ceske Budejovice only, where they also were relatively high in abundance and low in prevalence.

### Tick capillary artificial feeding with isolated bacteria

**Fig. 7:**
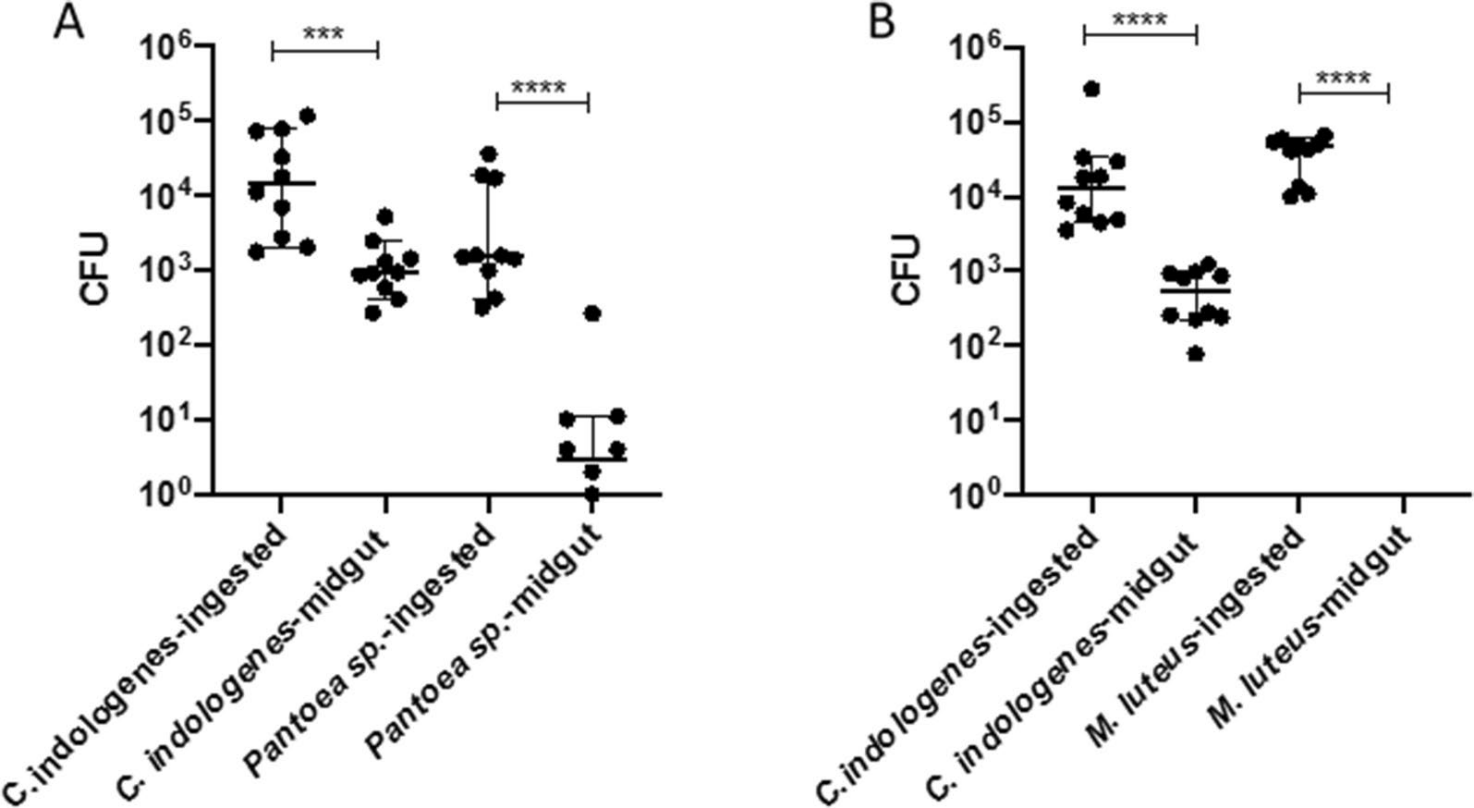
Colony-forming counts of bacteria ingested (in red) and isolated (in blue) from the midgut of the same individual of *Ixodes ricinus* adult females after 2 h of capillary feeding.** a** *Pantoea* sp.,** b** *Micrococcus luteus. Chryseobacterium indologenes* was used as a positive control. The results represent the median for 10 individual ticks. Asterisks indicate statistically significant difference at *** P < 0.001 and ****P < 0.0001

**Fig. 8:**
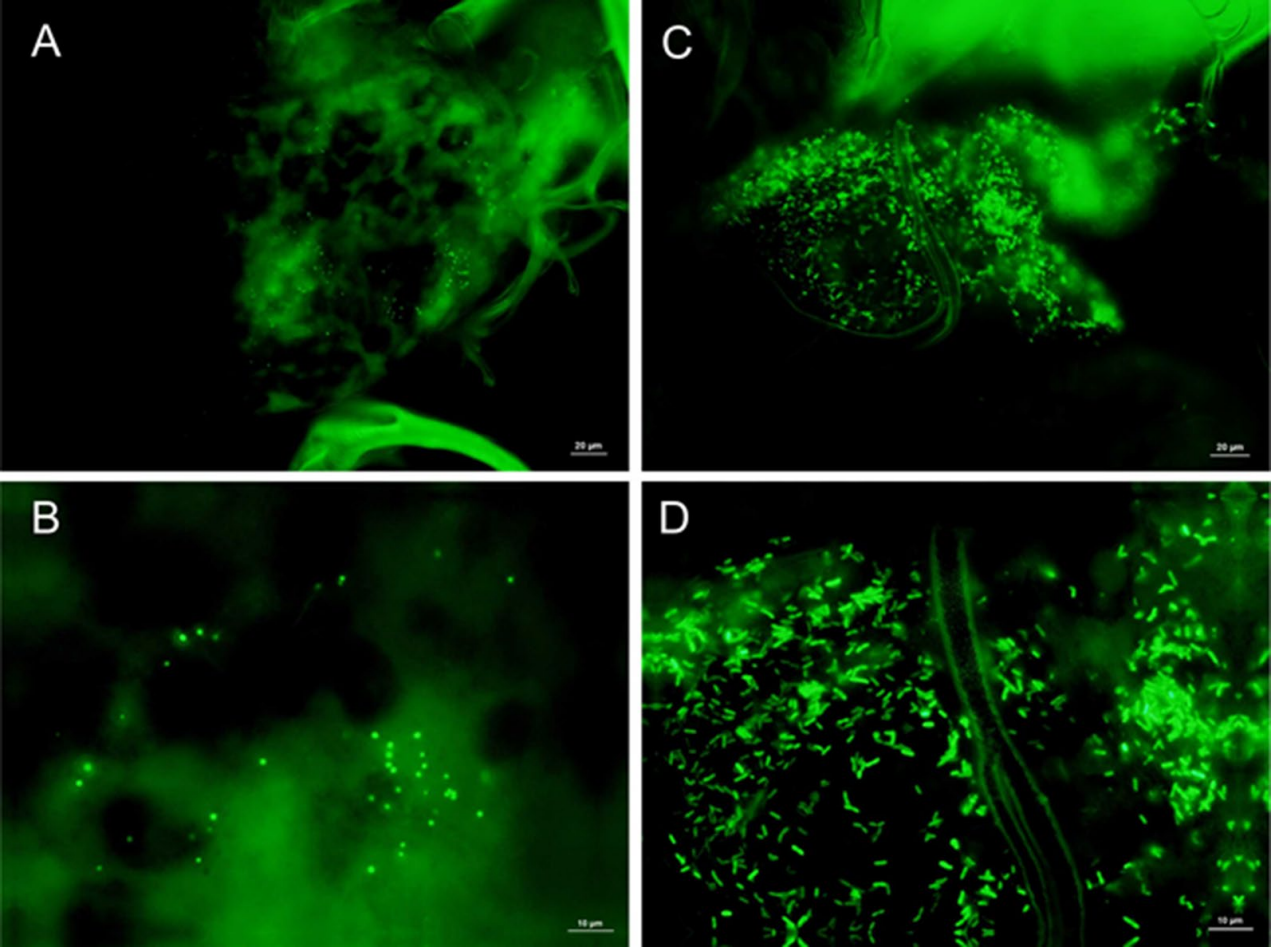
Fluorescent microscopy of capillary-fed GFP-labeled bacteria in the midgut of *Ixodes ricinus* adult females. **a**, **b** *Staphylococcus aureus*, **c**, **d** *Escherichia coli*. Abbreviations: GFP, Green fluorescent protein

The volume of the bacterial suspension uptake was converted into CFUs, resulting in an ingestion of between 10^2^ and 10^5^ cells per tick during 2 h of feeding. The analysis of ticks processed immediately after 2 h of feeding showed a great reduction of all bacterial taxa (Fig. 7a, b), with complete elimination of *M. luteus* (Fig. 7b). The positive control, *Chryseobacterium indologenes*, showed a much lower reduction compared to that of the two other bacteria. To visualize the cells in the midgut, we also fed a high concentration of GFP-labeled bacteria to the ticks, with these ticks ingesting between 10^8^ and 10^9^ cells per tick of GFP-labeled *E. coli* or GFP-labeled *S. aureus*. Microscopy revealed the presence of cells of both bacterial species in the tick midgut lumen (Fig. 8).

## Discussion

In a previous study, we used the culture-independent method and showed that the *I. ricinus* midgut microbiome is highly diverse but limited in abundance, and that it dramatically declined during blood-feeding [32]. In the current study, the culturable midgut bacterial microbiome of unfed *I. ricinus* females was low in terms of both prevalence and abundance. Only 37% of the analyzed midguts contained culturable bacteria, and among those, the abundance ranged from 2 to 1000 CFU/tick midgut (median: 9). Moreover, this microbiome was very diverse, with 32 species. Most of the species were isolated from only up to two individual ticks, indicating the absence of a culturable core microbiome. The majority of the identified bacterial taxa were typical representatives of the soil and plant environment or the mammalian skin, such as *Mycobacteroides, Micrococcus, Rhodococcus, Bacillus*,* Pseudomonas*,* Enterobacter*,* Streptococcus* and* Staphylococcus*, which in agreement with previous studies [25–28, 30, 31]. This result indicates that *I.* *ricinus* on occasion accidentally ingests bacteria from the environment. Although the culture-dependent approach applied here is limited to culturing aerobic and facultatively anaerobic bacteria growing on a broad-spectrum artificial medium, we believe it provides the evidence of low prevalence and abundance of extracellular bacteria in the midgut. Our results are in agreement with a low abundance and prevalence of culturable bacteria and the absence of a core microbiome in the midgut of other tick species, including *Ixodes scapularis* [49] and *Amblyomma americanum* [33], indicating that this feature might common across different ticks species and genera. Interestingly, this is in contrast to the highly abundant aerobic culturable gut microbiota of other blood-feeding arthropods, such as mosquitoes, analyzed under comparable cultivation conditions used in the current study [1–3]. It is also noteworthy that the majority of the culturable bacteria in the midgut of different tick species tend to be Gram-positive taxa [27, 30, 33].

To determine if the midgut bacterial community of *I. ricinus* is influenced by the environment, we compared ticks collected from two sites in the Czech Republic. Ticks from Brno were from an urban park, while ticks from Ceske Budejovice originated from a forest where there is relatively low human activity. The difference in alpha bacterial diversity based on the Shannon index was not statistically significant between the two localities; however, when the phylogeny of the identified taxa was considered, the two localities were significantly different based on Faith’s index. This shows that although the midgut microbiome was not influenced by the habitat based on the relative abundance, the bacterial community of the specific region tended to be phylogenetically more related than that of a different region.

The culture-independent approach revealed that the most prevalent bacterial taxa were known tick-borne pathogens and tick symbionts. The most abundant and prevalent genus was *Borrelia*, followed by the tick symbiont *Midichloria* sp. This is not surprising since the prevalence of the *Borrelia burgdorferi *sensu lato complex is high in the Czech Republic [50, 51]. Of interest was that in ticks with *Borrelia* sp., the prevalence of the other phyla was low. Similarly, *Rhipicephalus microplus* infected with *Theileria* sp. had an altered microbial composition, with a reduction in richness and evenness, referred to as a pathogen-induced dysbiosis [52]. It was also shown that *Anaplasma phagocytophilum* modified the *I. scapularis* microbiome via altering bacterial biofilm formation in the gut in order to infect the tick more efficiently [15]. These results suggest that tick pathogens can alter the native microbial community in the midgut. Overall, genera such as *Staphylococcus*, *Streptococcus*, *Ralstonia* and *Pelomonas* were relatively common, indicating an environmental influence on the tick midgut microbial community. As expected, several of the bacterial taxa found only by sequencing were not culturable under our laboratorial conditions, including *Borrelia*,* Spiroplasma*,* Midichloria* and *Rickettsia*. It is also likely that some of the bacterial taxa culturable under the conditions used in this study but which were detected by the culture-independent approach only represented DNA fragments of lysed/non-viable cells.

In order to investigate if the midgut microbiome can be manipulated to potentially negatively affect the tick vector competence for pathogens, we artificially fed adult females with indigenous bacteria isolated from the midgut. Capillary feeding is an established method to feed ticks with pathogenic and non-pathogenic bacteria [53, 54]. To test the reliability of our technique, we fed GFP-labeled Gram-negative (*E. coli*) or Gram-positive (*S. aureus*) bacteria to *I.* *ricinus* adult females. Cells from both species were visualized in the tick midgut 2 h after ingestion, confirming that the glass capillary feeding method was effective and that it is a suitable technique for in vitro bacterial feeding.

The indigenous *M. luteus* (Gram-positive) and *Pantoea* sp. (Gram-negative) were isolated from the *I. ricinus* midgut and used in the artificial feeding assays. *Pantoea* genus contains diverse species which are versatile in function and which have been previously isolated from *I. ricinus* [55–57]. *Microccocus luteus* is commonly found in soil, water and other environments, and it is also part of the mammalian skin microbiota. In other studies, *M. luteus* was cultured from *I. ricinus* in larvae [27] and nymphs [25]. Interestingly, *Micrococcus* spp. were also the most prevalent culturable taxon in *A. americanum* [33]. *Chryseobacterium indologenes*, the pathogen of the soft tick *Ornithodoros moubata*, was used as a positive control for bacterial ingestion and clearance [48]. Both *Pantoea* and *M. luteus* were rapidly cleared from the midgut within 2 h after ingestion, with complete elimination of *M. luteus*. A similar pattern of bacterial clearance was observed previously in *Dermacentor variabilis* capillary fed with *E. coli* and *Bacillus subtilis* [54]. In this study, although ticks ingested numerous bacterial cells, no CFUs could be cultured from the midgut after 3 h of feeding [54], suggesting a conserved general mechanism of bacterial clearance in the tick midgut. Taken together, these results led us to hypothesize that rapid reduction of bacteria in the *I. ricinus* midgut is the result of actions of the tick epithelial immunity and, during feeding, also of actions of antibacterial factors in the host’s blood. Clearly, further research into the molecular basis of bacterial clearance in the tick midgut is needed to improve our understanding of this process.

In conclusion, the results presented in this study show that the *I.* *ricinus* adult female midgut microbiome is poor in terms of abundance and prevalence, and that it is environmentally determined. An efficient and rapid bacterial clearance of extracellular bacteria by the midgut epithelial immunity appears to limit bacterial colonization in this organ although the mode of this phenomenon remains to be investigated.

## Supplementary Information

**Additional file 1: Table S1.** Abundance (log transformed) of bacterial genera detected in midgut of tick by the culture method.

## Data Availability

Not applicable.
